# Supplementary material for: Using machine learning and single nucleotide polymorphisms for improving rheumatoid arthritis risk Prediction in postmenopausal women
Source: PLOS Digit Health. 2025 Apr 9;4(4):e0000790. doi: 10.1371/journal.pdig.0000790 (PMC11981130; doi:10.1371/journal.pdig.0000790)
Supplement: Attachment — Submitted filename: response_letter_1008.docx [file pdig.0000790.s003.docx]

Dear Editor:

We very much appreciated the valuable comments provided by the reviewers. We have modified the manuscript accordingly and have included below point-by-point responses to the reviewers’ comments.

*Reviewer #1* *The study applies machine learning algorithms to Women's Health Initiative data, using SNPs and traditional risk factors to predict rheumatoid arthritis risk in postmenopausal women, achieving significant predictive accuracy. I have few points to raise*

1. *In Table 1, the correct figure is 40.5, not 405.*

Authors’ response: Thanks for the comment. I corrected the typo in this revision (Please see Table 1).

2. *Additional insights into the prevalence of the most influential SNPs are necessary, particularly noting the absence of overlap with SNPs commonly associated with RA, as identified in a prior literature (https://urldefense.com/v3/__https://www.ncbi.nlm.nih.gov/pmc/articles/PMC3030622/__;!!AU3bcTlGKuA!C-yYpvUjzabu70FoCvzLK3bXy-D9ihaNgEJYMEQZHEGp1qMX7NOBvhInwb1J5mTSZVkcVtwXYTelQncdug$ )*

Authors’ response: Thanks for your comments. The study you mentioned (Hughes LB, et al., 2010) was conducted among African Americans and focused on validating known RA risk alleles in that population. In contrast, we used SNPs from a different source (Yukinori Okada et al., 2014), a large-scale meta-analysis aimed at identifying new genetic risk factors and understanding RA biology across European and Asian populations, with a strong focus on therapeutic discovery. While Dr. Hughes’ study identified 27 SNPs significantly associated with RA, Dr. Okada’s study observed 101 SNPs, with only 5 SNPs (rs3087243, rs11889341, rs1980422, rs2476601, rs706778) overlapping between the two studies. Additionally, our study analyzed genome-wide SNPs, specifically focusing on postmenopausal women, which may explain the differences in the influential SNPs identified compared to Dr. Hughes' findings.

3. *The selection of phenotype variables was narrow, neglecting other potentially relevant factors such as the history of live births*

Authors’ response: Thanks for your insightful suggestion. In this revision, we acknowledged the limitation that only a narrow set of phenotype information is available and can be utilized in the present study due to lacking information in the existing data we can access. Broader phenotype risk factors will be explored in our future research. (Please see the 4^th^ paragraph of **Discussion**).

4. *It would be beneficial to observe the study's outcomes both before and after the resampling process.*

Authors’ response: Thank you for your valuable suggestion. Comparing the outcomes before and after the resampling process could provide additional insights. Given the complexity and potential for varied outcomes, conducting such an analysis could complicate the interpretation and shift the focus away from the primary objectives of our study. We plan to conduct additional research specifically focusing on methodological aspects for this comparison.

5. *The absence of a separate validation isn’t addresses particularly it seems that the hyperparameters were tuned on the test data.*

Authors’ response: Thank you for your comments. We acknowledge the importance of using a separate validation set for hyperparameter tuning. In our study, the test dataset is independent of training, we did not use testing data to turn hyperparameters. Instead, we used a cross-validation method for hyperparameter tuning within the training set. In the present study, the cross-validation approach within the training set provides a reliable framework for model tuning; and we use the independent testing dataset for validations.

*Reviewer #2: Major Comments:*

6. *Please provide some raw data and the relevant codes.*

Authors’ response: Thanks for your comments. To comply with the NIH Security Best Practices for Controlled-Access Data Subject to the NIH Genomic Data Sharing (GDS) Policy “Approved users must retain the original version of the encrypted data, track all copies or extracts and ensure that the information is not divulged to anyone except authorized staff members at the institution”, raw data can be only accessed by authorized and NIH-approved researchers. In the revision, we provide the details about data access and relevant codes (Please see the last sentence of the 1^st^ paragraph in the **Materials and Methods**).

7. *For verification of the results, please provide a link for the ML models developed after training along with a test data for verification.*

Authors’ response: Thanks for your comments. In this revision, we provide the relevant codes, all our preprocessing and model codes are available at https://github.com/68ngk3/RA_ML. In terms of data, to comply with the NIH Security Best Practices for Controlled-Access Data Subject to the NIH Genomic Data Sharing (GDS) Policy “Approved users must retain the original version of the encrypted data, track all copies or extracts and ensure that the information is not divulged to anyone except authorized staff members at the institution”, data can be only accessed with authorized and NIH-approved researchers (Please see the **Materials and Methods**).

8. *Please provide details of the Python libraries and the packages used.*

Authors’ response: Thanks for your comments. All statistical tests were two-sided, and the statistical significance was p < 0.05. Python programming language version 3.8.15 was used for all statistical analyses, utilizing NumPy, Pandas, Matplotlib, Seaborn, Scikit-learn, Xgboost, and Featurewiz were used in this study. We added the details of Python libraries in this revision (Please see the second to last sentence in **Statistical Methods**).

9. *Please provide supplementary data highlighting the actual phenotypic features and the Polygenic Risk Scores (PRS) features used for training various ML models.*

Authors’ response: Thanks for your comments. In the text, we provided the phenotype features of this study, please refer to “Well-established risk factors of RA were used in the current study, including age, race/ethnicity, physical activity, smoking status, and BMI, which were considered predictors”. In this revision, we added the details about the PRS (Please see the Genotype Information of **Materials and Methods**).

10. *Please provide details with respect to PRS data.*

Authors’ response: Thanks for your comments. The PRS was derived based on our previous study. More specifically, seven candidate scores were derived using different p-value thresholds (0.5, 0.4, 0.3, 0.2, 0.1, 0.05, and 0.001); the optimal PRS was selected based on the maximum area under the receiver operating characteristic curve (AUC) when predicting observed RA cases, and it was subsequently used for further analyses. In this revision, we added the details about the PRS (Please see the last sentence of Genotype Information under **Materials and Methods**).

11. *Please provide details regarding the self-reporting questionnaire used for data collection towards phenotypic features and methods employed to mitigate biases during the same.*

Authors’ response: Thank you for your insightful comment. In this revision, we have provided the link to the details of the questionnaire used in the study. The Women’s Health Initiative (WHI) is a large, comprehensive study, and the self-reporting questionnaire used for collecting phenotypic data was designed and validated through rigorous testing (Robert, et al., 2003). Specifically, the test-retest reliability of selected measures was evaluated in a subset of women from the Observational Study (OS) who participated in the Measurement Precision Study, ensuring the consistency of the responses over time to mitigate biases.

12. *Please provide details regarding MET and other calculations used in the study.*

Authors’ response: Thanks for your question. We provided the details regarding MET in Phenotype Information under **Materials and Methods**, please refer “The physical activity is normally expressed in metabolic equivalent of task (MET) units, and the expenditure of energy from recreational physical activity (MET-hours/week) was calculated based on questions about the participant’s usual activity exercise. The Physical Activity Guidelines for Americans 2008 suggested a minimum of 150 min/week of moderate-intensity exercise, which equals 7.5–14.9 MET-h/week. In this study, we categorized physical activity into four levels: no exercise (0); less than the guideline (0.1–7.4 MET-h/week); meeting the guideline (7.5–14.9 MET-h/week); and exceeding the guideline (≥15 MET-h/week)”.

13. *Please provide detailed information regarding data pre-processing and Feature*

Authors’ response: Thanks for your comments. In the text, we provided these following contents “Genotype imputation was conducted on the Sanger Imputation Server using the Haplotype Reference Consortium reference panel and Positional Burrows–Wheeler Transform imputation algorithm”, the missing value in phenotype were addressed by “the K-nearest neighbor algorithm was used for missing value imputation in phenotype information. The missing values were replaced with the mean values from similar or close neighbors, with a default number of neighbors (N=5)”. In this study, “Well-established risk factors of RA were used in the current study, including age, race/ethnicity, physical activity, smoking status, and BMI, which were considered predictors”. Additionally, for the genotype information, “Hybrid feature selection methods were utilized to address the dimensionality challenge caused by including whole genome-wide SNPs as individual predictors combining different feature selections in a multi-step process”（Please kindly refer the Genotype Information, Phenotype Information in **Materials and Methods** section）

14. *Engineering methods used before training the ML models.*

Authors’ response: Thanks for your comments. In this study, the missing values were replaced with the mean values from similar or close neighbors, with a default number of neighbors (N=5). Additionally, we identified and eliminated duplicate records to prevent biased model training. Then, outliers were found and removed from the dataset. Well-established risk factors of RA were used in the current study, including age, race/ethnicity, physical activity, smoking status, and BMI, which were considered predictors. SNPs with minor allele frequencies less than 0.01, a low P-value from the Hardy-Weinberg Equilibrium, or missing in a high fraction of subjects were filtered out. Pruning was performed with a window size of 200 variants, sliding across the genome with a step size of 50 variants at a time. SNPs with LD r2 higher than 0.25 were filtered out, and individuals with a first or second-degree relative in the sample were removed. Each SNP was coded as AA = 0, Aa = 1, aa = 2, implying that each additional copy of the minor allele increases the risk by the same amount. In this study, we categorized physical activity into four levels: no exercise (0); less than the guideline (0.1–7.4 MET-h/week); meeting the guideline (7.5–14.9 MET-h/week); and exceeding the guideline (≥15 MET-h/week). For the smoking status, women were classified as current, former, or never-smokers (participants who had not smoked 100 cigarettes in their lifetime). Former smokers were defined as answering ‘yes’ to the question, ‘Have you smoked 100 cigarettes in your life?’ but’ no’ to ‘Do you smoke cigarettes now?’ Current smokers were defined as answering ‘yes’ to both questions. The BMI was calculated based on measured weight and height. Individuals were categorized as underweight (BMI<18.5), normal weight(18.5≤BMI<25), overweight (25≤BMI<30), and obese (BMI≥30). Weight was measured in kg on a balance beam scale with the participant without shoes. Height was measured in 0.1 centimeters using a wall-mounted stadiometer. Hybrid feature selection methods were utilized to address the dimensionality challenge caused by including whole genome-wide SNPs as individual predictors combining different feature selections in a multi-step process 28. LASSO, a penalized regression method29, was utilized for SNP selection, and 4774 SNPs were retained based on their non-zero coefficient. Two methods were employed for dimension reduction; one is FeatureWiz, a quick and effective technique that uses Searching for Uncorrelated List of Variables (SULOV) algorithms and XGBoost to reduce features. Another one is kernel principal component analysis (KPCA) 30, which can construct nonlinear mappings that maximize the variance in the data. In this revision, we added the details by providing the relevant codes (Please see the **Materials and Methods**).

15. *Please provide details regarding the hyperparametric tuning performed.*

Authors’ response: Thanks for your comments. Grid search with cross-validation was utilized for the parameter tuning in this study, parameters were tuned based on the model characteristics. In this revision, we added the detail content and provided the relevant codes (Please see the Statistical Analysis of **Materials and Methods**).

16. *Kindly provide evidence to showcase that the baseline characteristics which significantly differ between the two cohorts aren’t contributing as confounding factors towards model building using genomic information.*

Authors’ response: Thanks for your comments. In this study, the models 2, 3, and 4 included the phenotype information (model 1). Thus, we clarified the details in this revision (Please see the **Statistical Analysis** of **Materials and Methods**).

17. *Kindly provide the results as a confusion matrix for the verification of the results.*

Authors’ response: Thanks for your comment. We found a typo on the figure 1, for the model 3 under logistic 3, the specificity should be 0.6 instead of 0.06, we corrected the figure in this revision. Since there are four matrices for each algorithm, we are unable to present all of them due to space constraints, we presented the confusion matrix of logistic regression here:

Model 1:

|  | Predicted + | Predicted - |
| --- | --- | --- |
| Actual + | 1248 | 889 |
| Actual - | 124 | 145 |

Model 2:

|  | Predicted + | Predicted - |
| --- | --- | --- |
| Actual + | 858 | 1287 |
| Actual - | 66 | 195 |

Model 3:

|  | Predicted + | Predicted - |
| --- | --- | --- |
| Actual + | 1416 | 764 |
| Actual - | 90 | 136 |

Model 4:

|  | Predicted + | Predicted - |
| --- | --- | --- |
| Actual + | 1420 | 700 |
| Actual - | 75 | 211 |

18. *Please provide the details of the methodology and the tool used for under-sampling.*

Authors’ response: Thanks for your comments. Under-sampling, which deletes most examples from the dataset so that the numbers of examples between different classes become balanced, was employed in our study. In this revision, we added the details about under-sampling. In terms of tools, we use the RandomUnderSampler from imblearn of Python (Please see the 2^nd^ sentence and last sentence of Statistical Analysis in **Materials and Methods**.

19. *Kindly provide the data as evidence towards improvement in F1 score upon integration of genomic information with traditional phenotypic predictors.*

Authors’ response: Thanks for your question. We revised the text to clarify this issue, thus Model 2=Model1+PRS, Model 3= Model 1+SNPs with FeatureWiz, Model 4=Model 1+SNPs with KPCA (Please see the **Statistical Analysis** of **Materials and Methods**).

20. *Kindly detail about the SNP selection methodology and the tool used.*

Authors’ response: Thanks for your question. In this revision, we added the details about SNPs selection, and we mentioned that PLINK 1.9 was utilized for this analysis (Please see the last sentence of Genotype Information under **Materials and Methods**)

21. *Please detail the methodology considering the reproducibility of the results.*

Authors’ response: Thanks for your comments. In this revision, we have included the relevant code, all our preprocessing and model codes are available at https://github.com/68ngk3/RA_ML, to ensure the reproducibility of our results. We have detailed the methodology, including all steps in the data preprocessing, model training, and evaluation phases in the code section, so that the results can be replicated. The provided code, along with clear instructions, will allow others to reproduce the analysis and validate the findings using the same dataset and parameters (Please see the **Materials and Methods**).

*Minor Comments:*

22. *Please provide details for DeLong Test and various model evaluation metrics used in the paper.*

Authors’ response: Thanks for your comments. In DeLong test, the z-score is calculated to assess the null hypothesis H0:AUC1=AUC2. The z-score measures the standardized difference between the two AUCs by considering their variances and covariance. In this revision, we added the details (Please see the **Statistical Analysis** under **Materials and Methods**).

We want to thank the reviewers for their comments and helpful suggestions. We believe these recommendations and subsequent revisions have greatly strengthened our manuscript and research work. We look forward to the editorial board’s ultimate decision regarding the acceptability of our manuscript.

Sincerely,

Qing Wu, M.D., Sc.D.

Professor

Department of Biomedical Informatics

College of Medicine

The Ohio State University
